# *Plectranthus amboinicus* and *Centella asiatica* Cream for the Treatment of Diabetic Foot Ulcers

**DOI:** 10.1155/2012/418679

**Published:** 2012-05-30

**Authors:** Yuan-Sung Kuo, Hsiung-Fei Chien, William Lu

**Affiliations:** ^1^Department of Surgery, National Taiwan University Hospital, Taipei 100, Taiwan; ^2^Department of Surgery, National Taiwan University College of Medicine, No. 1, Section 1, Jen-Ai Road, Taipei 10051, Taiwan; ^3^Oneness Biotech Co., Ltd., 7F-1, No. 3-1, Yuan Qu Street, Nangang District, Taipei 115, Taiwan

## Abstract

Effects of a topical cream containing *P. amboinicus* (Lour.) Spreng. (Lamiaceae) and *C. asiatica* (L.) Urban (Umbelliferae) were evaluated and compared to effects of hydrocolloid fiber wound dressing for diabetic foot ulcers. A single-center, randomized, controlled, open-label study was conducted. Twenty-four type 1 or type 2 diabetes patients aged 20 years or older with Wagner grade 3 foot ulcers postsurgical debridement were enrolled between October 2008 and December 2009. Twelve randomly assigned patients were treated with WH-1 cream containing *P. amboinicus* and *C. asiatica* twice daily for two weeks. Another 12 patients were treated with hydrocolloid fiber dressings changed at 7 days or when clinically indicated. Wound condition and safety were assessed at days 7 and 14 and results were compared between groups. No statistically significant differences were seen in percent changes in wound size at 7- and 14-day assessments of WH-1 cream and hydrocolloid dressing groups. A slightly higher proportion of patients in the WH-1 cream group (10 of 12; 90.9%) showed Wagner grade improvement compared to the hydrocolloid fiber dressing group but without statistical significance. For treating diabetic foot ulcers, *P. amboinicus* and *C. asiatica* cream is a safe alternative to hydrocolloid fiber dressing without significant difference in effectiveness.

## 1. Introduction

Development of foot ulcers is a common complication of diabetes. Approximately 15% to 20% of 16 million people with diabetes in the United States are hospitalized for foot ulceration and subsequent infection during the disease course [1]. Foot ulcers are primarily caused by peripheral neuropathy that reduces protective sensations for pain and temperature associated with trauma, and also by motor neuropathies that result in foot deformity, local pressure, and eventual ulceration [2]. A multicenter study identified peripheral sensory neuropathy, trauma and deformity as causative factors in 63% of diabetes patients; ischemia, callus formation, and edema were other prominent factors in ulcer etiology [3]. Infection often follows diabetic foot ulceration due to vascular insufficiency and reduced leukocyte function. Diabetic foot infections range from simple cellulitis to more complicated chronic osteomyelitis and are associated with increased frequency of infection, high morbidity with increased hospitalization, and lower extremity amputation [1, 4]. In diabetes patients, mild, moderate, and severe foot infections are all considered serious because the combination of an open wound and reduced immune defenses may quickly spread infection into the subcutaneous tissue and deeper structures [5]. Impaired microvascular circulation characteristic of diabetes limits transport of phagocytic cells and antibiotics to the affected area, predisposing diabetes patients to more frequent and potentially more severe foot lesions that are more difficult to treat. Treatment of foot ulcers and resultant infection may involve aggressive surgical debridement [4], oral or intravenous antibiotics [6, 7], and wound management with topical applications [8]. Metabolic abnormalities also require correction if foot ulcer treatment can be expected to be effective [2].

Prompt, effective treatment of foot ulcers is essential to reduce risk of exacerbation and amputation. Wound closure is the main treatment goal and the type of treatment employed depends on wound severity, vascularity, and whether infection is present. Treatment may include foot elevation, pressure relief, debridement of necrotic tissue, and application of various topical treatments, including wet-to-dry dressings, topical antiseptics, semipermeable films, foams, hydrocolloids, and calcium alginate swabs, among others [1]. Botanical and herbal treatments have been used for centuries in traditional Chinese medicine (TCM) as evidenced by recent reports of TCM drug use patterns in a general hospital in Taiwan [9]. We formulated an investigational botanical product designated as WH-1 cream for the treatment of diabetic foot ulcers. WH-1 cream contains extracts from two botanical raw materials, *Plectranthus amboinicus *(Lour) Spreng (Lamiaceae) and *Centella asiatica* (Linn) Urban (Umbelliferae). *P. amboinicus *and *C. asiatica* have exhibited anti-inflammatory and healing properties applicable to wound treatment. *P. amboinicus* is one of nearly 300 botanical species in the *Plectranthus* genus of the Lamiaceae family, which is noted for its diversity of use, particularly as medicines for skin, infective, digestive, and respiratory problems [10]. In its native environment in Kenya, Africa, it is the most frequent *Plectranthus* species applied to treat burns, wounds, sores, insect bites, and skin allergies. Evaluation of the *in vivo* (rat model) anti-inflammatory and antitumor activities of an extract from the leaves of *P. amboinicus* results showed significant reduction of edema and confirmed the anti-inflammatory properties at specific dosage levels [11]. In Taiwan and other East and South Asian countries, where *Plectranthus* species have been used for treating cough, fever, sore throat, mumps, and mosquito bites, Chiu et al. [12] investigated the anti-inflammatory and analgesic properties of *P. amboinicus* extract *in vivo* and *in vitro*. The extract inhibited pain induced in mice by acetic acid and formalin injection and inflammation induced by carrageenan. The anti-inflammatory effect was attributed to modulating antioxidant enzyme activity in the liver and production of tumor necrosis factor alpha (TNF-*α*). Shukla et al. [13] isolated asiaticoside from *C. asiatica* and found that hydroxyproline increased 56%, tensile strength increased 57% and both collagen and epithelialization increased after topical application of 0.2% asiaticoside solution to punch wounds in guinea pigs. The active constituents of *C. asiatica* include pentacyclic triterpene derivatives shown to be effective in treating venous insufficiency and striae gravidarum (pregnancy-related stretch marks) [14]. Its sedative, analgesic, antimicrobial, antiviral, and immunomodulatory effects have also been studied but without confirmation of specific effects.

To investigate the effectiveness of WH-1, we compared its effects to a relatively new hydrocolloid fiber dressing with demonstrated effectiveness (Aquacel Hydrofiber Dressing, ConvaTec, Valencia, CA, USA). This occlusive, adhesive dressing combines absorbent carboxy-methyl-cellulose nonwoven fiber to manage partial- and full-thickness wounds. It is reported to absorb up to three times its weight in exudate while providing an optimum healing environment and minimal bacterial cross-contamination [15]. Although it is one of the standard treatments applied by plastic surgeons, healing time results have varied [15–17].

While botanical and herbal therapies are usually applied safely and effectively, severe adverse effects of these agents have been reported when used either directly or combined with standard Western medical approaches [18, 19]. Therefore, such treatments must be subjected to scientific study to support their continued use by demonstrated safety and efficacy. This study aimed to evaluate the effects of a topical cream containing *P. amboinicus* and *C. asiatica* compared to the effects of hydrocolloid fiber wound dressing when applied topically to diabetic foot ulcers in patients with diabetes type 1 and type 2.

## 2. Materials and Methods

A single-center, randomized, controlled, open-label study was conducted between October 2008 and December 2009.

### 2.1. Subjects

Twenty-four type 1 or type 2 diabetes patients aged 20 years and older with Wagner grade 3 foot ulcers postsurgical debridement were enrolled. Wagner grade 3 was defined as “deep ulcer involving osteitis, abscess, or osteomyelitis” according to the Wagner classification system [20].

Inclusion criteria were patients with type 1 or type 2 diabetes, aged 20 years or older, and having Wagner grade 3 foot ulcers postsurgical debridement. Patients with poor nutritional status (albumin <3 g/dL), poor diabetic control (HbA1c >10%), anemia (hemoglobin <10 g/dL), and leukocyte counts <1,000/cu mm were excluded. Other exclusion criteria were presence of connective tissue disease or known or suspected malignancy local to the study ulcer; renal failure insufficiency (serum creatinine >1.5 mg/dL) or abnormal liver function (AST, ALT >2.5 × upper limit of normal range); requiring treatment with immunosuppressive agents, corticosteroids, chemotherapy or radiotherapy; female patients with positive pregnancy test or breastfeeding or unwilling to use appropriate contraceptive methods during study; patients with known sensitivity to essential oils or lanolin cream. Withdrawal criteria included withdrawal of consent, lost to follow up, treatment failure (defined as worsening of Wagner grade or serious infection in ulcer site), and requiring the use of a prohibited medication. Withdrawal was also a consideration if any other medical condition was present that, in the opinion of the principal investigator, indicated that continued treatment with study therapy and participation in this trial was not in the best interest of the patient. The 24 patients were randomly assigned to two equal groups comprising 12 patients in a WH-1 cream group treated with WH-1 cream containing *P. amboinicus* and *C. asiatica* twice daily for two weeks and another 12 patients in a hydrocolloid group treated with hydrocolloid fiber dressings during the same time period.

### 2.2. Ethical Considerations

All enrolled patients were advised about their participation in the study and all provided signed informed consent. The study protocol was reviewed and approved by the Institutional Review Board (IRB) of National Taiwan University Hospital (IRB no. 200801067 M).

### 2.3. Formulation of Investigational Product: WH-1 Cream

WH-1 cream contained extracts from two botanical raw materials, *P. amboinicus* and *C. asiatica*. The plants of *P. amboinicus* were collected on 2007 according to good agricultural and good collection practices. *C. asiatica*. extract was sourced commercially with certificate of analysis of the extract and herbal material. Guided by previous pharmacological wound healing studies [11–14], the most active fractions, PA-F4 from *P. amboinicus* and S1 from *C. asiatica,* were combined in a 1 : 4 ratio to form the drug substance WH1-DS. The final drug product, WH-1 cream, contained 1.25% of drug substance in a cream base, 15 g per tube. The cream base contained cetostearyl alcohol, ireine, liquid petrolatum, methyl paraben propyl paraben, Span 60, Tween 60, white petrolatum, water, and pigments.

### 2.4. Treatment Administration

During the two-week study period, WH-1 cream was applied topically twice daily to 12 WH-1 group patients in an amount to fully cover the ulcer area in a thin and even layer. The maximum amount must not exceed 2 millimeters in thickness. After each application, the comparative product, hydrocolloid fiber wound dressing (AQUACEL Hydrofiber Dressing) was applied to 12 patients in the hydrocolloid fiber dressing group and left in place for up to 7 days or changed earlier as clinically indicated. After applying WH-1 cream or hydrocolloid fiber dressing, the wound was covered with a transparent, adhesive, waterproof dressing (Opsite, Smith & Nephew, Taipei, Taiwan). After wound dressing for two weeks, the wounds in both groups were all reconstructed by split-thickness skin graft or primary closure.

 Treatment allocation was performed before site initiation. Permuted-block treatment allocation was used to assign participants to each group. A list of sequential numbers was generated using a permuted-block randomization procedure with a block size of 4 in SAS 9.1, with each number randomly assigned to one group. Patients meeting the inclusion and exclusion criteria were randomly assigned in a 1 : 1 ratio to the WH-1 cream group or the hydrocolloid fiber dressing group according to a predefined randomization schedule. After random assignment, subjects received the study medication at the baseline visit and were assessed for wound condition and safety during the 2-week treatment period (follow-up visits at the 7th and 14th day). Hydrocolloid fiber dressing group patients had the dressing applied at baseline and changed at the 7th day or earlier if clinically indicated.

### 2.5. Wound Assessment

Prior to beginning the study, all patients in both treatment groups had received surgical debridement with complete reduction of necrotic tissue and debris to visualization of bleeding healthy tissue. After the study treatment period, the primary endpoint was the percentage change in wound size defined as wound size at endpoint (days 7 and 14) compared to wound size at baseline (postsurgical debridement). The secondary endpoint was the improvement in Wagner Grade from baseline (all patients were Wagner grade 3 at baseline postdebridement) to final visit at day 14. Safety assessment involved the observation of presence or absence of adverse events for the study period.

Laboratory parameters measured included: hemoglobin, HbA1c, WBC with differential counts, AST, ALT, albumin, creatinine, fasting plasma glucose, BUN, and urine protein, which were tested at screening/baseline and at the end of study (day 14). A pregnancy test was indicated for females of child-bearing potential before the study began.

Physical examination and vital signs were done at baseline and at days 7 and 14. Concomitant treatments were permitted as follows: sharp surgical debridement (including resection of necrotic soft tissue and bone, sinus tracts, fistulae, undermined borders, callus) to form viable wound margins was performed before randomization and repeated as needed during the dosing period. Systemic antimicrobial agents were allowed for treatment of infections. Nonweight bearing or offloading was required for all subjects. Prohibited treatments during the study period included immunosuppressive agents, corticosteroids, chemotherapy and radiotherapy.

### 2.6. Statistical Analysis

Demographic parameters and other baseline characteristics were analyzed by treatment group for the randomized population. The intent-to-treat (ITT) population contained all randomized patients who had received at least one dose of study medication and had at least one follow-up efficacy endpoint evaluation. Additionally, only patients with a Wagner grade 3 foot ulcer were included in the ITT population. Analysis of primary and secondary endpoints was based on the ITT population. The safety population was defined as all randomized patients who had taken any dose of study medications and was the primary population for analyzing the safety data.

Comparability among the two treatment groups was evaluated using Mann-Whitney *U* test for continuous variables and Fisher's exact test for categorical variables. Data are presented as a median (interquartile range) for continuous data and numbers (percentages) for categorical data. The Wilcoxon signed ranks test was used to compare differences before and after treatment in each group. All statistical assessments were two-sided and evaluated at the 0.05 level of significant difference. Statistical analyses were performed using SPSS 15.0 statistics software (SPSS Inc., Chicago, IL, USA) and SAS 9.1 (SAS Institute Inc., Cary, NC, USA).

## 3. Results

**Figure 1:**
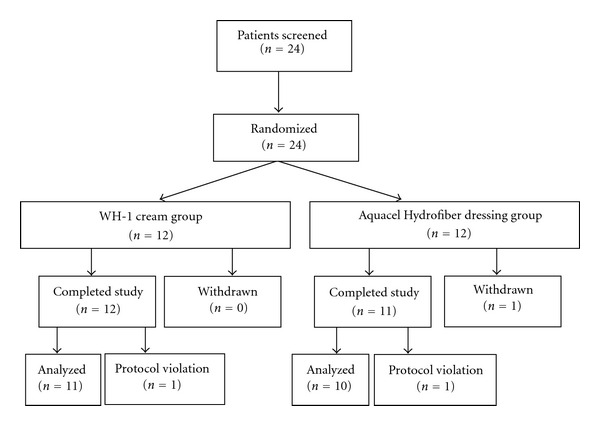
Patients' flow chart.

A total of 24 patients were screened and enrolled into the study between October 29, 2008 and December 14, 2010. Of these, 12 patients were randomly assigned to the WH-1 cream group and 12 patients to the hydrocolloid fiber dressing group. No participants were excluded during screening.  Figure 1 shows a flow chart of the trial that presents the reasons for early termination and protocol violations in detail. Only one patient in the hydrocolloid fiber dressing group did not complete the study because the patient withdrew consent and decided to receive full-thickness skin grafts. Two other patients (1 in WH-1 group and 1 in hydrocolloid fiber dressing group) were protocol violating because their ulcers were not Wagner grade 3 at enrollment.

WH-1 cream group patients included 4 men and 8 women with a median age of 75.5 years (range, 36–87 years) and the hydrocolloid fiber dressing group included 5 men and 7 women with a median age of 69.5 years (range, 44–89 years). No statistically significant differences were found between the two groups in patients' demographics and baseline characteristics (*P* > 0.05) (Table 1).

**Figure 2:**
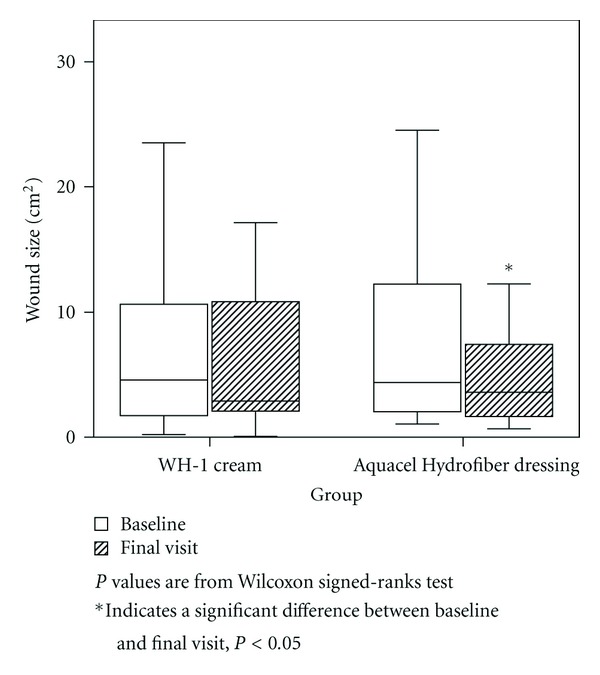
Changes of wound size.

The ITT population comprised 21 patients; 11 patients treated with WH-1 and 10 patients treated with hydrocolloid fiber dressing. The primary endpoint, percent changes in wound size from baseline at the final visit (day 14, end week 2) in the ITT population are shown in Table 2. No statistically significant differences were found in the percent changes in wound size between the WH-1 cream and hydrocolloid fiber dressing groups (*P* = 0.673). Analysis of results within treatment groups showed that only the hydrocolloid fiber dressing group had significant decreases in wound sizes from baseline to the final visit (*P* = 0.037, Figure 2).

**Figure 3:**
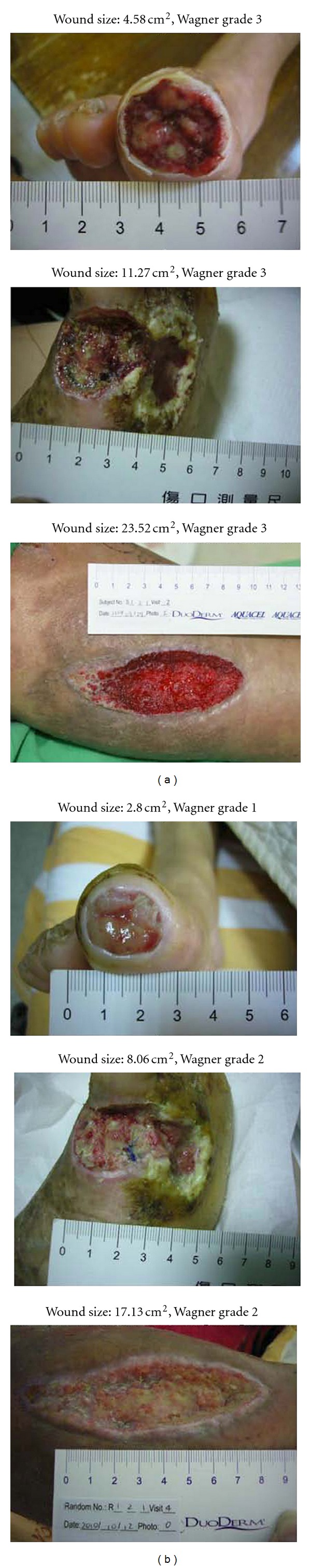
The improvement in wound size and Wagner grades of three patients in the WH-1 cream group. (a) Before WH-1 cream treatment, (b) the 14th day after treatment.

After 2 weeks of treatment, 4 (36.4%) patients had Wagner grades 2 and 6 (54.6%) patients had Wagner grade 1 in the WH-1 cream group; 4 (40.0%) patients had Wagner grade 2 and 3 (30.0%) patients had Wagner grade 1 in the hydrocolloid fiber dressing group. The secondary endpoint was the proportion of patients who had improvements in Wagner grade, and improvement in the WH-1 group (10 of 11 patients, 90.9%) was slightly higher than that in the hydrocolloid fiber dressing group (7 of 10 patients, 70.0%), but the difference was not statistically significant (*P* = 0.311).  Figure 3 shows photographs demonstrating improvements in wound size and Wagner grades of the WH-1 cream group.

All adverse events (AEs) were collected by patients or monitored by the investigator throughout the study and were coded using the 13th quarter Medical Dictionary for Regulatory Activities (MedDRA version 13.1). The numbers (%) of patients with at least one adverse event of maximum severity during the treatment period are summarized in Table 3. During the treatment period, 5 of 12 patients in the WH-1 cream group (41.7%) and 5 of 12 patients in the hydrocolloid fiber dressing group (41.7%) reported at least one adverse event. According to the investigator's judgment, none of these events was study treatment related. No significant difference in adverse events was found between the WH-1 cream and hydrocolloid fiber dressing groups (*P* > 0.05). In addition, there was no death or serious adverse events in any patient during this clinical study.

## 4. Discussion

In this study, we compared the effects of a botanical cream containing *P. amboinicus* and *C. asiatica* with the effects of hydrocolloid fiber wound dressing when applied topically to diabetic foot ulcers. No statistically significant differences were found in the percent changes in wound size and improvements in Wagner grade between two groups of diabetes patients who were treated with either WH-1 cream or hydrocolloid fiber dressings for the two-week study period. Although the proportion of patients who showed improvement in Wagner grade was slightly higher in patients treated with WH-1 cream than in those treated with hydrocolloid fiber dressing (90.9% versus 70%, resp.), the difference was not significant. However, within-group analyses showed that only the hydrocolloid fiber dressing group had significant decreases in wound sizes from baseline to the final visit. Safety assessment was similar in both groups, with only minimal adverse events and no serious adverse events reported or observed during the study period. Also, no pronounced changes were seen in mean SBP, DBP, pulse rate and body temperature in any patient. Few changes were noted in clinical laboratory data (i.e., hematology, serum chemistries, and urinalysis) and none were clinically significant.

Taking Chinese herbs is a common self-treatment behavior in Asian society as well as a frequently prescribed treatment, especially for patients with major diseases [9]. It is not unusual that we apply a botanical medicine to the treatment of diabetic foot ulcers and achieve good results without adverse events. However, reports of severe adverse effects induced by botanical or herbal agents directly or by combined use with standard Western medicine are found in the literature [18, 19]. Systemic absorption of those agents remains problematic. In this study, results showed that the systemic absorption of WH-1 cream was minimal and it appeared to be safe and well tolerated when administered to patients with diabetic foot ulcers. Our results provide helpful evidence and insight into the use of extracts from Chinese herbs for modern treatment applications.

The insignificant differences between WH-1 and hydrocolloid fiber dressing can be explained in part because of the relatively short observation period in this preliminary study. Most short-term studies showed preliminary “healing” signals such as reepithelization, which can only be confirmed through advanced confirmatory research. For example, “Regranex” (becaplermin, the only FDA-approved prescription platelet-derived growth factor gel for deep neuropathic diabetic foot ulcers) demonstrated only a slight improvement in ulcer volume (*P* = 0.056) when used in 41 patients in a 28-day dose-ranging phase II study [21]. However, Regranex was reported to have a 50% incidence of complete wound closure in the pivotal 20-week phase III study [22]. Other studies of topical applications for foot ulcers have been longer but have not necessarily yielded better results. For example, in a comparative study between honey and povidone iodine applied as dressing solutions for Wagner grade 2 diabetic foot ulcers, patients were followed for 6 weeks and a mean healing time of 14.4 days (range 7 to 26 days) was achieved for patients receiving honey treatment compared to 15.4 days (range 9–36 days) for povidone iodine patients; even without significant difference in healing time, it could still be concluded that honey was a safe alternative dressing for Wagner grade 2 foot ulcers [23]. Another multicenter trial investigating three dressings for managing diabetic foot ulcers had a duration of 12 weeks and significant differences were noted in results between the three treatment groups but none directly related to mean healing time [15]. Nevertheless, in future research expanding our present preliminary study results, we will apply longer continuous treatment and observation of diabetic foot ulcers.

Another possible explanation for lack of significant differences between WH-1 cream and a topical cream with a hydrocolloid preparation could be the comparison of “apples and oranges.” However, the hydrocolloid wound dressing used in our study (Aquacel Hydrofiber Dressing) was one of three dressings investigated in a large, multicenter trial to determine whether modern dressings were more clinically effective than traditional dressings in the treatment of diabetes-related foot ulcers [15]. In that “apples to apples” comparison, no differences in effectiveness were found in the three dressings in terms of percentage healed at 24 weeks or in the mean time to healing or quality of healing (i.e., incidence of recurrence). While that study compared parallel treatments over a longer period of time, the only significance was that the hydrocolloid fiber dressing proved to be significantly more costly per treatment even though fewer changes of dressing were required to achieve the same results. This suggests that more information about the effectiveness of WH-1 could be gained through comparisons with a wider range of topical medicines as well as newer products in widespread use.

Further investigation of *P. amboinicus* and *C. asiatica* cream must include other clinical parameters such as pulsation, Doppler ultrasound, and patients' subjective symptoms. Also, in the present study, only wound sizes and improvements in Wagner grades were compared and other signs and symptoms associated with diabetic foot ulcers, including pain, numbness, pallor, and pulses, were not compared. We also did not focus on infection of foot ulcers since our investigational product was not intended as an antimicrobial measure; however, infection was noted and recorded in some patients and systemic antimicrobial agents were allowed for treatment of infections. When diabetic foot ulcers of different Wagner grades and infection by different organisms were investigated by culture and sensitivity and susceptibility to citric acid as the sole antimicrobial agent, citric acid gel was 94% effective in controlling foot infections for Wagner grades 1 and 2 and also in Wagner grade 3 without deep osteomyelitis [8]. One drawback of the Wagner classification system is that it does not factor in the presence of infection [4]. Since infection may be another factor in the insignificant results between the investigated WH-1 cream versus a highly absorbent hydrocolloid fiber dressing, future studies may expand our approach to determine possible relationships between infection, wound sizes, and improvements in Wagner grade.

## 5. Limitations

This study has certain limitations, including that it is a single-site study with a small sample and a short observation period for wound reconstruction. It is essentially a pilot study conducted to observe safety and effectiveness of the botanical cream designated as WH-1 in treating Wagner grade 3 foot ulcers since the investigated product is currently undergoing a phase II clinical trial for treatment of Wagner grade 1 diabetes foot ulcers [http://clinicaltrials.gov/ct2/show/NCT00709514?term=wh1&rank=1]. Future study will include comparisons with a wider range of topical treatments for diabetic foot ulcers and an expanded sample and longer study period.

## 6. Conclusion

For treating diabetic foot ulcers, *P. amboinicus* and *C. asiatica* cream is a safe alternative to hydrocolloid fiber wound dressing without significant differences in effectiveness. *P. amboinicus* and *C. asiatica* cream is a safe and effective alternative for use in patients for whom hydrocolloid fiber wound dressing may be contraindicated.

## Figures and Tables

**Table 1:** Demographics and baseline characteristic of patients with Wagner grade 3 foot ulcers (*n* = 24).

	WH-1 cream (*n* = 12)	AQUACEL Hydrofiber dressing (*n* = 12)	*P* value
Age (years)^1^	75.5 (50.0, 82.0)	69.5 (59.0, 79.5)	0.862
Gender, *n* (%)^2^			1.000
Male	4 (33.3)	5 (41.7)	
Female	8 (66.7)	7 (58.3)	
Target side, *n* (%)^2^			
Right	5 (41.7)	6	
Left	7 (58.3)	6	
Height (cm)^1^	160 (144, 166)	160 (151, 168)	0.758
Weight (kg)^1^	64 (50, 101)	67 (63, 77)	0.758
BMI (kg/m^2^)^1^	30.82 (24.01, 36.68)	25.04 (22.97, 30.68)	0.356
Wound size (cm^2^)^1^	3.92 (1.18, 10.64)	4.47 (2.57, 14.57)	0.386

*P* values are from ^1^Mann-Whitney *U* test and ^2^Fisher's exact test.

Values are ^1^median (interquartile) and ^2^number (percentage).

**Table 2:** Efficacy issue-primary and secondary endpoints in intent-to-treat population (*n* = 21).

	WH-1 cream (*n* = 11)	AQUACELHydrofiber dressing (*n* = 10)	*P* value
Primary endpoint			
Percent changes in wound size (%)^1^	−27.18 (−38.86, 36.10)	−22.64 (−36.90, −3.20)	0.673
Secondary endpoint			
Improvement on Wagner Grade, *n* (%)^2^	10 (90.9)	7 (70.0)	0.311

*P* values are from ^1^Mann-Whitney *U* test and ^2^Fisher's exact test.

Values are ^1^median (interquartile) and ^2^number (percentage).

**Table 3:** Summary of adverse events (*n* = 24).

	WH-1 cream (*n* = 12)	AQUACELHydrofiber dressing (*n* = 12)	*P* value
Any adverse event(s)	5 (41.7%)	5 (41.7%)	1.000
Constipation	2 (16.7%)	3 (25.0%)	1.000
Tinea pedis	22 (16.7%)	0 (0.0%)	0.478
Hypoglycemia	1 (8.3%)	0 (0.0%)	1.000
Dizziness	1 (8.3%)	0 (0.0%)	1.000
Visual field defect	1 (8.3%)	0 (0.0%)	1.000
Conjunctivitis	0 (0.0%)	1 (8.3%)	1.000
Diarrhoea	0 (0.0%)	1 (8.3%)	1.000
Gastritis	0 (0.0%)	1 (8.3%)	1.000
Vomiting	0 (0.0%)	1 (8.3%)	1.000
Oedema	0 (0.0%)	1 (8.3%)	1.000

*P* values are from Fisher's exact test.

## Abbreviations

AE: Adverse events
IRB: Institutional Review Board
ITT: Intent to treat
TNF-*α*: Tumor necrosis factor alpha
TCM: Traditional Chinese medicine.
